# Contained rupture of a sinus of Valsalva aneurysm: Is it just a matter of luck?

**DOI:** 10.1186/s13019-022-01800-2

**Published:** 2022-03-28

**Authors:** Giorgio Vigano, Rozemarijn Vliegenthart, Daniël K. M. Pollack, Massimo A. Mariani

**Affiliations:** 1 Department of Cardiothoracic Surgery, Heart Centre, University of Groningen, University Medical Center Groningen, P.O. Box 30.001, 9700 RB Groningen, The Netherlands; 2 Department of Radiology and Nuclear Medicine, University Medical Centre Groningen, Groningen, The Netherlands

**Keywords:** Contained aortic rupture, Aortic root replacement, Acute aortic syndrome, Bicuspid aortic valve

## Abstract

**Background:**

Contained rupture of the ascending aorta is a rare condition, but the severity of this complication enforces strict guidelines for its prevention and a prompt diagnosis, once already occurred.

**Case presentation:**

A 66-year-old man with a history of type 2 diabetes, longstanding aortic valve stenosis and aortic root aneurysm of 47 mm was hospital admitted for elective surgery. A Bentall-De Bono procedure was performed in order to replace the stenotic bicuspid aortic valve and exclude the dilated portion of the aortic wall. Intraoperatively, a discontinuity of the aortic wall, just above the aortic annulus, at the non-coronary sinus of Valsalva was incidentally observed. The aortic wall discontinuity was none other than a contained aortic rupture. The preoperative CT-scan images were afterwards analyzed by the radiologist, in order to identify the contained aortic rupture. Indeed a false aneurysm of the non-coronary sinus of Valsalva of a maximum diameter of 15 mm was detected, thanks to a 3D reconstruction.

**Conclusions:**

The diagnosis of contained aortic rupture is certainly demanding, particularly in absence of signs or symptoms of rupture in a chamber of the heart or in the pericardium. Although this case represents a consensus of experts’ opinion, the recognition of these specific cases in which the risk of dissection, rupture or death is at its highest, would allow to operate at the appropriate time, improving the outcomes.

## Background

A spontaneous contained aortic rupture is a rare, but life-threatening condition [1]. The diagnosis of a contained aortic rupture is always demanding, since it shows no specific imaging findings and the signs and symptoms are primarily related to the site of the rupture, into the heart chambers, pericardial cavity or pulmonary artery [2]. A contained aortic rupture should always be suspected, in presence of an aortic aneurysm concomitantly detected with specific conditions, such as a pericardial effusion, a right atrial mass, or a continuous cardiac murmur [1–3]. We present herein a case of a completely asymptomatic contained aortic rupture, in which the operation was successfully performed and luck certainly played a role.

## Case presentation

**Fig. 1:**
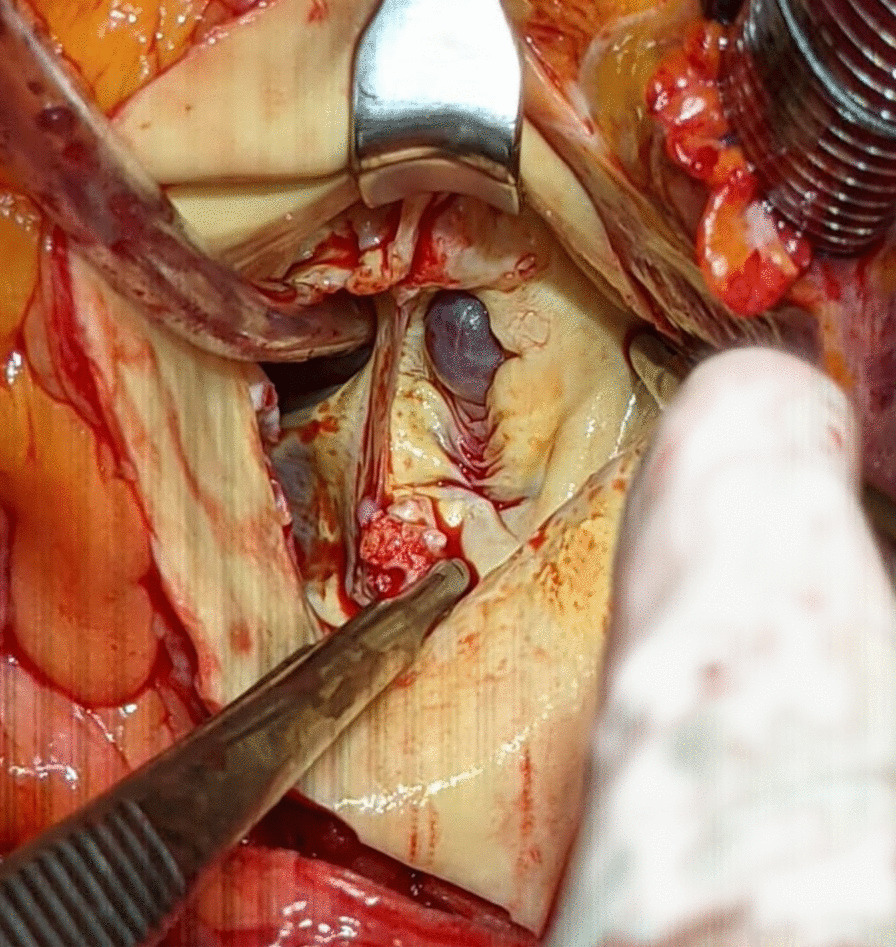
Intraoperative findings

**Fig. 2:**
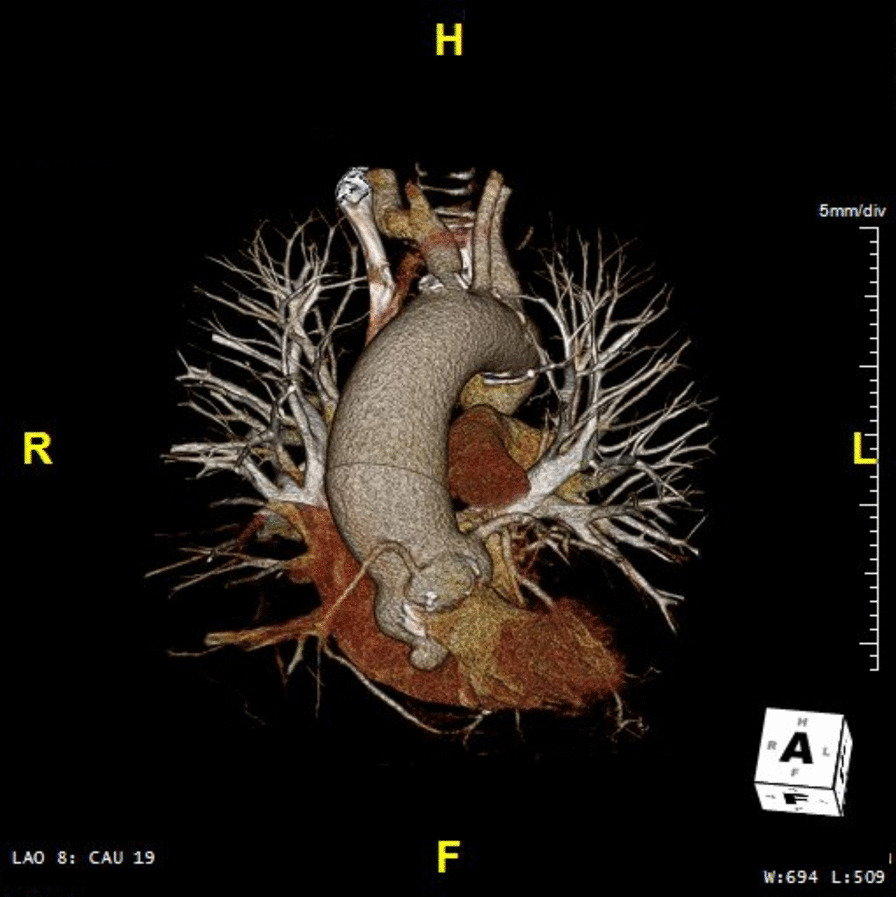
3D CT-scan reconstructions

**Fig. 3:**
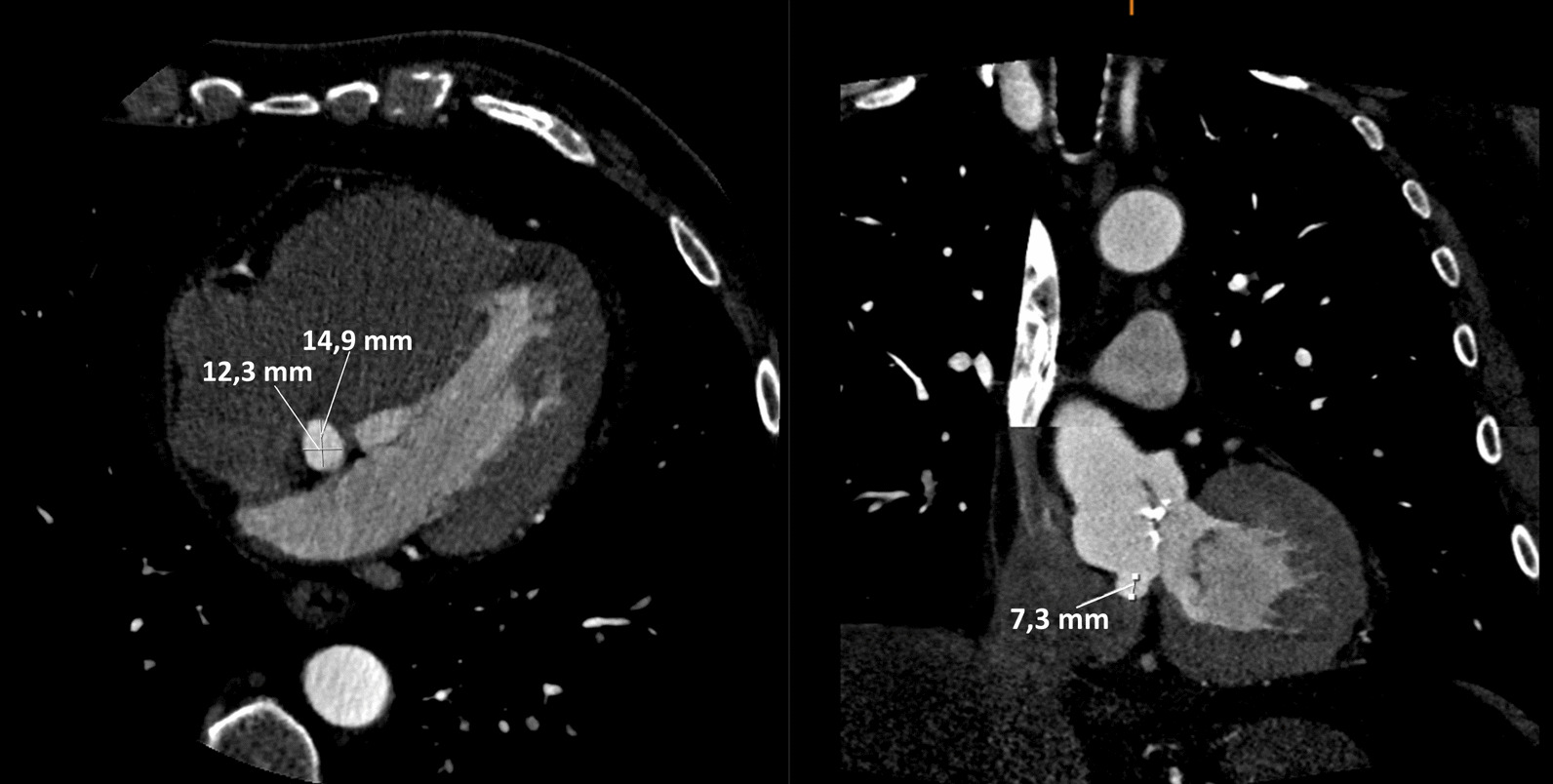
CT-scan measurements of the false aneurysm (contained aortic rupture)

A 66-year-old man with a history of type 2 diabetes and a bicuspid aortic valve, was discussed in the heart team, owing to a longstanding severe aortic valve stenosis. A CT-scan was preoperatively performed and showed a moderate dilatation of the aortic root with a maximum diameter of 47 mm at the level of the aortic root and ascending aorta. No obstructive coronary artery disease was seen at the coronary angiography and the patient was accepted and scheduled for an elective aortic valve and root replacement. Intraoperatively, once the ascending aorta was opened, a discontinuity of the aortic wall, above the annulus, at the Valsalva non-coronary-sinus was observed (Fig. 1). In absence of any communication neither with the right atrium, nor with any other cavity, this condition was classified as a contained aortic rupture, which. was successfully excluded during a biological aortic valve, root and ascending aorta replacement according to Bentall-De Bono. At the light of the intraoperative findings, an accurate analysis of the preoperative CT-scan and a 3D reconstruction (Fig. 2), showed a non-coronary sinus of Valsalva false aneurysm of a maximum diameter of 15 mm (Fig. 3), in absence of contrast media extravasation. An informed consent was obtained and the patient was genetically screened for familial thoracic aortic aneurysm and dissection (familial TAAD) and connective tissue disorders. Histopathological examination of the aortic wall showed no abnormalities of any kind. The patient was hospital discharged on the twelfth postoperative day, after an unremarkable postoperative course, unless a transitory atrioventricular conduction disturbance, spontaneously recovered.

## Discussion and conclusions

The primary purpose of the surgical replacement of the aortic root and ascending aorta is to prevent the related adverse events, such as rupture, dissection and death. Aortic diameter is a major criterion for recommending elective operation in asymptomatic patients. This assumes that the risk of an adverse event exceeds the risk of elective operation for diameters greater than 55 mm. Patients with Marfan syndrome or other genetically mediated disorders (e.g. bicuspid aortic valve) should undergo elective operation at smaller diameters, owing to the underlying cellular disorders, that make the aortic wall weaker and therefore these patients more prone to acute dissection or rupture [4]. For patients with an indication for aortic valve surgery, an aortic diameter ≥ 45 mm is considered to indicate concomitant surgery of the aortic root or tubular ascending aorta [5]. Although in our case the international recommendations were fulfilled, the surgical correction was performed behind time. Thank the luck, the rupture of the aortic wall had no serious consequences for the patient, since it remained “contained” and no extravasation or bleeding occurred. We can only hypothesize that the adverse event didn’t acutely occurred and that, a normal blood pressure level could have favourably played a role. Although this case represents a consensus of experts’ opinion, the accurate recognition of these specific cases in which the risk of dissection, rupture or death is at its highest, would dramatically improve the outcomes. While the “appropriate” aortic diameter for intervention represents a topic for discussion, it is still debated in which case of aortic root dilatation, additional examinations (such as 3D reconstruction) would be justified or necessary, to define the urgency of the surgery and to minimize the risk of catastrophic events (from acute rupture). In our specific case the patient had no recent medical history of inflammatory diseases, infections or trauma, therefore no apparent reason for more complex preoperative work-up. A contained rupture of the ascending aorta urges strict guidelines and a prompt recognition, in order to prevent and eventually correct this life-threatening condition.

## Data Availability

The datasets used and/or analysed during the current study are available from the corresponding author on reasonable request.
